# Supplementary material for: EGCG Prevents the Onset of an Inflammatory and Cancer-Associated Adipocyte-like Phenotype in Adipose-Derived Mesenchymal Stem/Stromal Cells in Response to the Triple-Negative Breast Cancer Secretome
Source: Nutrients. 2022 Mar 5;14(5):1099. doi: 10.3390/nu14051099 (PMC8912398; doi:10.3390/nu14051099)
Supplement: Supplementary file 1 [file nutrients-14-01099-s001.zip › Legends and figures for Supplemental 1 and Supplemental 2.pdf]

Supplemental.1

A.

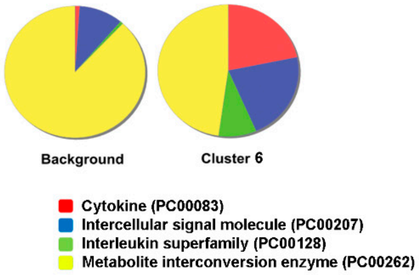

B.

PANTHER Protein Class	Fold Enrichment	FDR
Cytokine	100	1.23E-06
Intercellular signal molecule	11.43	8.10E-03
Interleukin superfamily	67.66	4.03E-02
Metabolite interconversion enzyme	2.88	4.54E-02
**GO Molecular Function**		
Cytokine activity	23.60	1.04E-03
Cytokine receptor binding	12.53	1.60E-02

Figure S1. Enrichment analysis of the DEGs in Cluster 6. (**A**) Pie charts of protein class variation after a GO enrichment analysis showing the behavior of background genes and genes clustered in 6. (**B**) Fold enrichment values for protein class and molecular functions with a FDR ≤0.05 as a cut off, and using as background all genes detected.

Supplemental.2

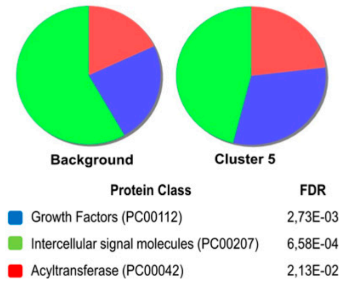

Figure S2. Gene ontology (GO) enrichment analysis results of genes from cluster 5. Pie chart of protein class variation with a false discovery rate (FDR) ≤0.05 as a cut off, and using all genes detected as background.
